# Subacute Sensory Ataxic Neuronopathy With Thymoma Presenting Marked Improvement After Steroid Therapy

**DOI:** 10.3389/fneur.2019.00268

**Published:** 2019-03-20

**Authors:** Haruo Nishijima, Ken Onodera, Nobuyuki Sato, Tatsuya Ueno, Hiroki Hikichi, Rie Haga, Akira Arai, Chieko Suzuki, Jin-ichi Nunomura, Masayuki Baba, Masahiko Tomiyama

**Affiliations:** ^1^Department of Neurology, Aomori Prefectural Central Hospital, Aomori, Japan; ^2^Department of Thoracic Surgery, Aomori Prefectural Central Hospital, Aomori, Japan

**Keywords:** nerve conduction study, paraneoplastic neurological syndrome, subacute sensory ataxic neuronopathy, steroid, thymoma

## Abstract

Subacute sensory ataxic neuronopathy is a well-known form of paraneoplastic syndrome. Most sensory neuronopathies are associated with small cell lung cancer and anti-Hu antibodies, and usually show only slight improvement with immunotherapy. To date, there have been few reports of neuropathy associated with thymoma and no treatment strategy has been established for thymoma-related neuropathy. Here, we provide the first report of a case of sensory ataxic neuronopathy with thymoma that showed marked improvement after steroid therapy, even though preceding intravenous immunoglobulin treatments and tumor resection were less effective. A 57-year-old Japanese man was referred to our hospital with a 6-week history of distal paresthesia in his four limbs and an unsteady gait. He presented with left-dominant ataxia in his four limbs due to reduced sensation in his extremities. He also complained of constipation, difficulty urinating, and erectile dysfunction. Upon investigation, including electrodiagnostic studies, the patient was diagnosed as having sensory ataxic neuronopathy with invasive thymoma. A first round of intravenous immunoglobulin therapy, a following thymectomy, and a second round of intravenous immunoglobulin therapy after the surgery were not effective in treating his neurological symptoms. Subsequently, oral steroid therapy was started, which brought about a remarkable improvement; 6 weeks after the beginning of the steroid therapy, his neurological symptoms were resolved, except for slight distal paresthesia in his feet. Although rarely reported, thymoma can underlie sensory neuronopathy, and the response of thymoma-associated sensory neuronopathy to immunotherapy might be better than that of anti-Hu antibody-related neuropathies. Even if the first immunotherapy is not effective in treating neuropathy with thymoma, further immunomodulatory treatment should be tried after treating the tumor.

## Background

Subacute sensory ataxic neuronopathy is a widely-known form of paraneoplastic syndrome (PNS) and is considered to be one of the “classical syndromes” (1). The tumor that most frequently underlies sensory neuronopathy is a small cell lung cancer, and patients with this cancer usually present with anti-Hu antibodies (2). The prognosis for paraneoplastic neuropathy differs depending on the underlying tumors and antibodies presented by the patients (3). For subacute sensory neuropathy associated with a tumor, immunomodulatory or immunosuppressant treatments sometimes provide a slight improvement or stabilization of neurological symptoms, but the results are inconclusive (2). For patients with anti-Hu antibodies, treatment of the cancer was the only factor associated with the stabilization of neurological symptoms (4).

There have been a handful of reports of neuropathy associated with thymoma (5–9), but as yet a treatment strategy has not been established for thymoma-related neuropathies. As far as we are aware, only one report to date has described a patient with sensory ataxic neuronopathy with thymoma, with the patient showing a remarkable neurological improvement after resection of the thymoma and intravenous injection of immunoglobulins (IVIg) (9). Sensory neuropathy with thymoma might be more likely than anti-Hu antibody-associated PNS to respond to immunotherapy. In this report, we present the first case of sensory ataxic neuronopathy with thymoma that showed a marked improvement after steroid therapy, although preceding IVIg treatments and tumor resection were less effective. Our case suggests that immunotherapy can be beneficial for neuropathy with thymoma, even if the first trial is ineffective.

## Case Presentation

**Figure 1:**
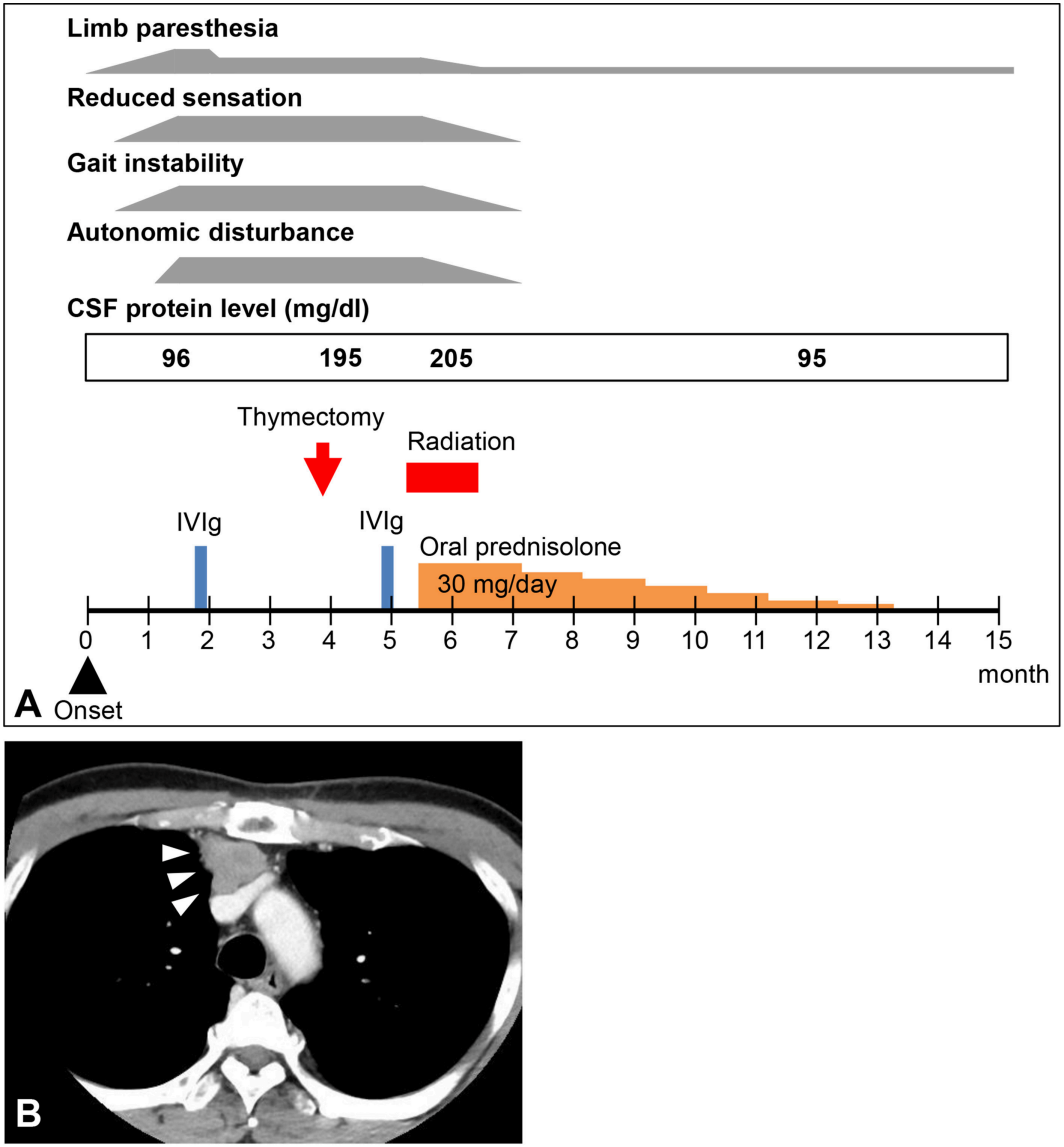
**(A)** Clinical course of the patient. CSF, cerebrospinal fluid; IVIg, intravenous immunoglobulin. **(B)** Computed tomography image of the patient's chest. A mediastinal tumor was detected (arrowheads).

A 57-year-old Japanese man was referred to our hospital with a 6-week history of distal paresthesia in his four limbs and unsteady gait (Figure 1A). He was an office worker with a medical history of diabetes mellitus and hyperuricemia. He had no family history of neurological disorders. On admission, physical examination revealed no abnormalities. Neurologically, he presented with normal cranial nerve function except for impaired taste sensation, and normal strength in all four limbs, although clumsiness was observed in both hands due to reduced sensation. The nose-to-finger test and the heel-knee test revealed left side-dominant mild ataxic movements in all four limbs, which were worsened by eye-closing. The patient had paresthesia in his four extremities. Touch sensation was disturbed in all four distal limbs and pain sensation was reduced in both hands, but vibration sensation was preserved. Position sensation was disturbed in both feet. Deep tendon reflexes were absent, apart from a reduced response in his right quadriceps femoris. He needed a cane while walking, and his walking appeared ataxic because he used a wide-based gait in a careful manner; the Romberg sign was positive. The patient complained of constipation, urination difficulty, and erectile dysfunction. Blood studies revealed hyperglycemia (192 mg/dl; normal range 70–139 mg/dl) with an HbA1c level of 6.1% (normal range 4.6–6.2%) and hypertriglyceridemia (296 mg/dl; normal range 30–149 mg/dl). Tumor markers were within normal levels, except for an elevated squamous cell carcinoma antigen level of 9.8 ng/ml (normal range < 1.5 ng/ml). Anti-nuclear antibody was positive (titer, 1:160). Anti-GM1 IgM antibody was positive at low titer (0.118 optical density; normal range < 0.1). Onconeural antibodies, including anti-amphiphysin, CV2, PNMA2 (Ma2/Ta), Ri, Yo, Hu, recoverin, SOX1, titin, zic4, glutamic acid decarboxylase of 65 kDa, and Tr (delta notch-like epidermal growth factor-related receptor) antibodies were all negative. Other autoantibodies, including anti-acetylcholine receptor, ganglionic acetylcholine receptor, and anti-neutrophil cytoplasmic antibodies were all under normal limits. Antimyelin-associated glycoprotein and anti-ryanodine receptor 1 antibodies were not examined. Angiotensin-converting enzyme and lysozyme were within normal levels. Immunoelectrophoresis revealed no abnormal M-protein. Cerebrospinal fluid examination revealed an elevated protein level (96 mg/dl; normal range 20.0–40.0 mg/dl), with very slight pleocytosis (6 cells/mm^3^; normal range < 5 cells/mm^3^). The IgG index was within normal levels (0.69; normal range < 0.7). Cytological analysis of cerebrospinal fluid showed no malignancy. Magnetic resonance imaging of the whole spine showed no spinal canal stenosis or abnormal intensity change in the spinal cord. Gadolinium enhancement was seen in some parts of the cauda equina and lumbar plexus.

The results of electrodiagnostic studies are shown in Table 1. Motor distal latencies of the bilateral median nerves were prolonged. Sensory nerve action potentials were slightly decreased, as were sensory nerve conduction velocities. Repetitive nerve stimulation study was performed in the right ulnar nerve and the compound muscle action potentials were recorded from the abductor digiti minimi muscle, revealing no abnormal waning or waxing. Somatosensory-evoked potentials were not examined. Eight weeks after onset of neurological symptoms, IVIg was commenced under the tentative diagnosis of chronic inflammatory demyelinating polyradiculoneuropathy, which slightly improved distal paresthesia in the four limbs. After IVIg, neurological symptoms were stabilized; however, reduced sensation and gait instability persisted and remained unimproved. Subsequent whole-body computed tomography revealed a tumorous lesion in the anterior mediastinum (Figure 1B). Sixteen weeks after the onset of neurological symptoms, the patient underwent a thymectomy. Microscopic examination revealed thymoma, classified as type B3, with invasion of the right brachiocephalic vein.

**Table 1:** Results of the nerve conduction study prior to thymoma resection.

**Motor**	**DL (ms)**	**CMAP (D/P) (mV)**	**MCV (m/s)**	**F-Lat (ms)**
Right median nerve	6.2^*^	3.9/3.6	55.0	30.1
Right ulnar nerve	3.5^*^	7.8/7.7	52.9	32.3^*^
Right tibial nerve	5.8	9.2/6.9	50.7	52.5
Left median nerve	6.3^*^	3.7/3.5	52.8	31.4^*^
Left ulnar nerve	4.0^*^	8.1/7.7	47.1^**^	31.3
Left tibial nerve	5.4	12.7/9.1	36.8^**^	55.3
**Sensory**	**SNAP (D/P) (μV)**		**SCV (D/P) (m/s)**	
Right median nerve	14.7^**^/7.8^**^		42.6^**^/49.3^**^	
Right ulnar nerve	25/32		44.0^**^/57.6	
Right sural nerve	5.7		45.2	
Left median nerve	14^**^/10^**^		39.2^**^/51.4^**^	
Left ulnar nerve	21/12^**^		38.7^**^/44.4^**^	
Left sural nerve	9.5		44.8	

* *over the normal limit*;

** *below the normal limit*.

*CMAP, compound muscle action potential; D, distal; DL, distal latency; F-Lat, minimum F-wave latency; MCV, motor nerve conduction velocity; P, proximal (above elbow in ulnar nerves); SCV, sensory nerve conduction velocity; SNAP, sensory nerve action potential*.

*Normal values: Median Nerve DL < 4.2, CMAP > 3.5, MCV > 48, F-Lat < 31; Ulnar Nerve DL < 3.4, CMAP > 2.8, MCV > 49, F-Lat < 32; Tibial Nerve DL < 6.0, CMAP > 2.9, MCV > 41, F-Lat < 58; Median Nerve SNAP (D/P) > 19/>16, SCV (D/P) > 44/>53; Ulnar Nerve SNAP (D/P) > 18/>14, SCV (D/P) > 47/>54; Sural Nerve SNAP > 3.8, SCV > 39*.

For 4 weeks after the surgery, the patient's neurological symptoms remained unimproved and he still needed a cane while walking. The results of electrodiagnostic studies at this time are shown in Table 2. Sensory nerve action potentials were further decreased compared with those of the first study. A second round of IVIg was then commenced, and at 5 weeks after surgery radiation therapy for thymoma was started. There was no neurological relief at 6 weeks after the surgery (22 weeks after the onset of neurological symptoms), so oral steroid therapy (prednisolone, 30 mg/day) was started. He showed remarkable improvement of neurological symptoms 1 week after the steroid therapy started and 6 weeks after the steroid therapy started, the patient's neurological symptoms, including decreased sensation, hand clumsiness, gait instability, and autonomic failure, were resolved, except for slight distal paresthesia in his feet. The oral dose of prednisolone was gradually reduced and ended 32 weeks after it commenced, with no re-aggravation of his symptoms (Figure 1A). The results of electrodiagnostic studies 28 weeks after the start of steroid therapy are shown in Table 3. The results from his motor nerves were completely normal and sensory nerve action potentials were increased compared with the results of the pre-steroid examination.

**Table 2:** Results of the nerve conduction study after thymoma resection and before steroid therapy.

**Motor**	**DL (ms)**	**CMAP (D/P) (mV)**	**MCV (m/s)**	**F-Lat (ms)**
Right median nerve	5.1^*^	9.0/8.2	51.3	36.6^*^
Right ulnar nerve	3.5^*^	8.1/7.3	54.1	36.8^*^
Right tibial nerve	5.6	12.1/7.9	39.6^**^	60.7^*^
Left median nerve	5.5^*^	7.8/7.0	51.3	35.9^*^
Left ulnar nerve	3.5^*^	6.0/5.7	47.2^**^	35.3^*^
Left tibial nerve	4.5	9.8/6.8	43.2	54.7
**Sensory**	**SNAP (D/P) (μV)**		**SCV (D/P) (m/s)**	
Right median nerve	5.4^**^/4.3^**^		48.3/54.1	
Right ulnar nerve	9.1^**^/6.6^**^		42.9^**^/51.4^**^	
Right sural nerve	6.4		38.3^**^	
Left median nerve	2.9^**^/1.4^**^		36.6^**^/54.1	
Left ulnar nerve	7.0^**^/6.3^**^		46.3^**^/51.3^**^	
Left sural nerve	3.5^**^		34.0^**^	

* *over the normal limit*;

** *below the normal limit*.

*CMAP, compound muscle action potential; D, distal; DL, distal latency; F-Lat, F-wave latency; MCV, motor nerve conduction velocity; P, proximal (above elbow in ulnar nerves); SCV, sensory nerve conduction velocity; SNAP, sensory nerve action potential*.

*Normal values: Median Nerve DL < 4.2, CMAP > 3.5, MCV > 48, F-Lat < 31; Ulnar Nerve DL < 3.4, CMAP > 2.8, MCV > 49, F-Lat < 32; Tibial Nerve DL < 6.0, CMAP > 2.9, MCV > 41, F-Lat < 58; Median Nerve SNAP (D/P) > 19/>16, SCV (D/P) > 44/>53; Ulnar Nerve SNAP (D/P) > 18/>14, SCV (D/P) >47/>54; Sural Nerve SNAP >3.8, SCV > 39*.

**Table 3:** Results of the nerve conduction study after steroid therapy.

**Motor**	**DL (ms)**	**CMAP (D/P) (mV)**	**MCV (m/s)**	**F-Lat (ms)**
Right median nerve	3.3	10.4/9.4	50.0	27.4
Right ulnar Nerve	2.9	8.0/7.6	56.6	28.0
Right tibial nerve	4.4	14.8/7.3	44.6	47.1
Left median nerve	3.7	11.2/10.8	53.1	28.3
Left ulnar nerve	3.1	7.5/7.3	58.5	28.9
Left tibial nerve	3.2	11.3/6.1	43.4	48.8
**Sensory**	**SNAP (D/P) (μV)**		**SCV (D/P) (m/s)**	
Right median nerve	13.3^**^/5.6^**^		47.8/63.6	
Right ulnar nerve	11.4^**^/3.8^**^		51.8/63.7	
Right sural nerve	5.8		47.5	
Left median nerve	13.1^**^/5.9^**^		47.8/74.5	
Left ulnar nerve	6.0^**^/4.3^**^		50.0/63.8	
Left sural nerve	11.8		48.7	

** *below the normal limit*.

*CMAP, compound muscle action potential; D, distal; DL, distal latency; F-Lat, F-wave latency; MCV, motor nerve conduction velocity; P, proximal (above elbow in ulnar nerves); SCV, sensory nerve conduction velocity; SNAP, sensory nerve action potential*.

*Normal values: Median Nerve DL < 4.2, CMAP > 3.5, MCV > 48, F-Lat < 31; Ulnar Nerve DL < 3.4, CMAP > 2.8, MCV > 49, F-Lat < 32; Tibial Nerve DL < 6.0, CMAP > 2.9, MCV > 41, F-Lat < 58; Median Nerve SNAP (D/P) >19/>16, SCV (D/P) > 44/>53; Ulnar Nerve SNAP (D/P) > 18/>14, SCV (D/P) > 47/>54; Sural Nerve SNAP > 3.8, SCV > 39*.

## Discussion

We provide the first report of a case of thymoma-related sensory ataxic neuronopathy that showed good responsiveness to steroid therapy. The present case exhibited subacute sensory ataxic symptoms. Although we diagnosed our patient as having “probable” sensory neuronopathy according to the diagnostic criteria proposed by Camdessanche et al. (10), it was difficult to distinguish between sensory neuronopathy and axonal sensory neuropathy from the first evaluations. In this case, the reduction of sensory nerve action potentials was slight at the first examination (Table 1), although sensory disturbance was apparent. Sensory symptoms remained stable, but sensory nerve action potentials had decreased severely by the second examination (Table 2). This suggested that dorsal root ganglia were primarily affected. Furthermore, in our patient, the sensory nerves in his upper extremities were affected to a greater extent than those in his lower extremities, as shown by electrodiagnostic studies (Tables 1–3), suggesting non length-dependent sensory neuropathy. Thus, we diagnosed him as having sensory neuronopathy rather than axonal sensory neuropathy. However, we acknowledge that some of his sensory symptoms and electrophysiological abnormalities may have been due to a concomitant diabetic neuropathy.

Although he did not show muscle weakness clinically, motor nerve conduction studies revealed prolonged distal latencies and prolonged minimum F-wave latencies (Tables 1, 2). Tajima et al. also reported a case of predominant sensory ataxic neuronopathy presenting with prolonged motor distal latencies (9). Prolonged motor distal latencies were a common feature detected by electrodiagnostic studies in both these cases. More case studies are warranted to confirm whether this electrophysiological feature is characteristic of thymoma-related sensory neuronopathy.

Our patient clinically presented with typical symptoms of subacute sensory neuronopathy, which is well established as a “classical syndrome” of PNS. He had B3-type thymoma and met the diagnostic criteria for PNS (11). However, the possibility that the thymoma was just a coincidental lesion with neuropathy could not be completely ruled out. His neurological symptoms showed no improvement 6 weeks after tumor resection. Treatment of thymoma alone did not affect his neurological symptoms, at least during the follow-up period presented here. Remarkable improvement of neurological symptoms was found only after the start of steroid therapy. Thus, it is possible our patient incidentally had both thymoma and an autoimmune neuropathy, such as chronic inflammatory demyelinating polyradiculoneuropathy. Biopsy of a peripheral nerve or dorsal root ganglion would have been useful to exclude autoimmune neuropathies other than PNS; however, we did not perform a nerve biopsy because of its invasiveness. Nevertheless, thymectomy alone might have been effective at the long term follow-up, because it was possible to completely withdraw the steroid therapy without symptom relapse. There were only 6 weeks between the thymectomy and the start of prednisolone therapy, so we cannot exclude a possible effect of thymoma removal on patient recovery.

Only treatment of tumors has shown apparent efficacy in resolving PNS-related sensory neuronopathy, although the effect is sporadic and heterogeneous (2, 4, 12). Immunomodulatory treatments are only sometimes effective in showing a slight improvement or stabilization of neurological symptoms (2, 4, 12). There have been some reports on thymoma-related neuropathy in the literature. Among them, the case reported by Tajima et al. showed neurological improvement following a second round of IVIg after thymoma resection (9). Neuronopathy with thymoma might respond better to immunotherapy than classical PNS-related sensory neuronopathy presenting with small cell lung cancer and anti-Hu antibodies. Thus, even if the first immunotherapy is not effective in treating neuropathy with thymoma, further immunomodulatory treatment should be tried after tumor treatment. More specifically, sensory ataxic neuronopathy with thymoma may respond well to IVIg, steroids, or both, but more studies that include a larger number of patients are needed to confirm this idea.

Our patient presented with anti-GM1 IgM antibody positivity. This antibody is characteristic of multifocal motor neuropathy, which is one of the autoimmune neuropathies, and steroid therapy sometimes worsens this type of neuropathy (13). However, the anti-GM1 IgM antibody is not specific for multifocal motor neuropathy and can test positive in other dysimmune neuropathies or motor neuron disease, especially when the titer is low, as it was in the present case (13). Thus, the clinical significance of this antibody in the present case is uncertain.

## Conclusion

This case demonstrates three important points: (1) although rarely reported, thymoma can underlie sensory neuronopathy; (2) sensory neuronopathy with thymoma might respond better to immunotherapy than neuronopathy with small cell lung cancer and anti-Hu antibodies; (3) prolonged distal latencies from the motor nerve conduction studies may be associated with the thymoma-related neuropathy, even if clinical features were restricted to sensory and autonomic symptoms.

## Data Availability

The datasets generated for this study are available on request to the corresponding author.

## Ethics Statement

Written informed consent was obtained from the patient for the publication of this case report.

## Author Contributions

HN: study concept, patient care, data collection, analysis and interpretation of data, and writing the manuscript; KO, HH, TU, RH, AA, CS, JN, MB, NS, and MT: patient care, data collection, and review of the manuscript.

### Conflict of Interest Statement

The authors declare that the research was conducted in the absence of any commercial or financial relationships that could be construed as a potential conflict of interest.
